# Repeated abortion in adulthood induces cognition impairment in aged mice

**DOI:** 10.1038/s41598-018-29827-3

**Published:** 2018-07-30

**Authors:** Lili Wang, Ying Zhang, Haofeng Wang, Hui Li, Ziying Zhao, Ning Wang, Bin He, Cuige Shi, Shucheng Zhang, Jiedong Wang

**Affiliations:** 1 Graduate School of Peking Union Medical College, Beijing, China; 2 Department of Cell Biology, National Research Institute for Family Planning, Beijing, China; 3 Department of Neurology, The Fifth People’s Hospital of Jinan, Jinan, China; 4 Department of Anatomy, Capital Medical University, Beijing, China; 5 Department of Chinese Medicine, Air Force General Hospital, Beijing, China

## Abstract

Age-related cognitive decline is one of the major aspects that impede successful aging in humans. Repeated abortion in adulthood can accelerate or aggravate cognitive deficiency during aging. Here we used repeated abortion in female mice adulthood and investigated the consequences of this treatment on cognitive performance during aging. We observed a substantial impairment of learning memory in 15 months old. This cognitive dysfunction was supported by Aβ elevation in CA region. Repeated abortion mice have uniform estrous cycles and decreased ERα expression in hypothalamus and hippocampus. Furthermore, repeated abortion not only significantly increased the HMGB1 expression in hippocampus but also increased the plasma and hippocampal protein levels of IL-1β, IL-6, and TNF-α. Finally, we identified that MPP-induced cell apoptosis and increased HMGB1 expression as well as IL-1β, IL-6, and TNF-α expression as following Aβ elevation. Taken together, our results identify possible molecular mechanisms underlying cognitive impairment during aging, and demonstrated the repeated abortion in adulthood on cognitive function in aged mice.

## Introduction

Cognitive psychologists study things that people do in their heads and how they subsequently perform based on those mental operations^1^. Approximately half of all women who have an abortion have had one or more previous abortions. Women with a history of more than one abortion are likely to suffer more severe physical and psychological problems after abortion. A perspective views abortion as a potentially stressful life event within the range of other normal life stressors^2^. Derived from psychological theories of stress and coping, this perspective emphasizes that because abortion occurs in the context of a second stressful life event-a pregnancy that is unwanted, unintended, or associated with problems in some way - it can be difficult to separate out psychological experiences associated with abortion from psychological experiences associated with other aspects of the unintended pregnancy^3^. Much of the public debate over abortion and mental health has framed the question as follows: Does abortion cause harm to women’s motivation and spatial cognition?

In recent years, estrogen receptors have increasingly been identified as involved in modulating motivation and cognition in female human development, postmenopausal mood disorders, and corresponding animal models^4–6^. ERα are present in male and female brains. There is evidence that cognitive deficits can be rescued by estrogens^7^. Most studies focused on sexual and aggressive behavior^8^. The evidence of estrogenic effects on neuronal plasticity, is contrasted by only a few studies on the effects of more general states such as motivation and mood and their outcome in behavioral performance^9,10^. Furthermore, stress axis activity have been reported to be affected by estrogenic mechanisms in male mice and rats^11,12^. Estrogenic effects in learning and memory have also been reported in both sexes. Among the classical features of aging in humans, we find cognitive impairment, dementia, memory loss, etc^13^. As estrogen levels change with age, especially in females, it is important to know the effects of low estrogen level on ERα distribution on learning and memory^14^.

In the present study, we examined the effect of repeated abortion on the cognition responses in aging. This cognitive dysfunction was supported by Aβ elevation. We also determined changes in estrous cycle and ERα expression in various brain regions. Furthermore, we found that HMGB1 and neuroinflammatory involved in the learning and memory impairment observed *in vivo* and *in vitro*. Therefore, the study offers the possibility to address the intriguing question of whether repeated abortion in adulthood may impact on cognition in the elderly.

## Materials and Methods

### Animals

Kunming mice (18–22 g) were used. Animals were housed with free access to food and water at a mean ± SD constant temperature of 22 ± 2 °C, humidity of 55 ± 5%, and a 12 h light/12 h dark cycle. This study was performed in strict accordance with the recommendations in the Guide for the Care and Use of Laboratory Animals of the National Institutes of Health. The protocol was approved by the Committee on the Ethics of Animal Experiments of the National Research Institute of Family Planning.

### Pregnancy determination

Mice were caged at 17:00 (male: female = 2:1), and the vaginal plug were checked at 8:00 in the next day. Seeing the vaginal plug was set as 0.5 days of pregnancy. Mice weighted 6.5 days after seeing the vaginal plug. It was determined pregnancy and follow-up tests if the mice body weight increased more than 1 g. Furthermore, it was thought not pregnant and given up follow-up tests if the mice body weight increased less than 1 g.

### Control model

**Figure 1:**
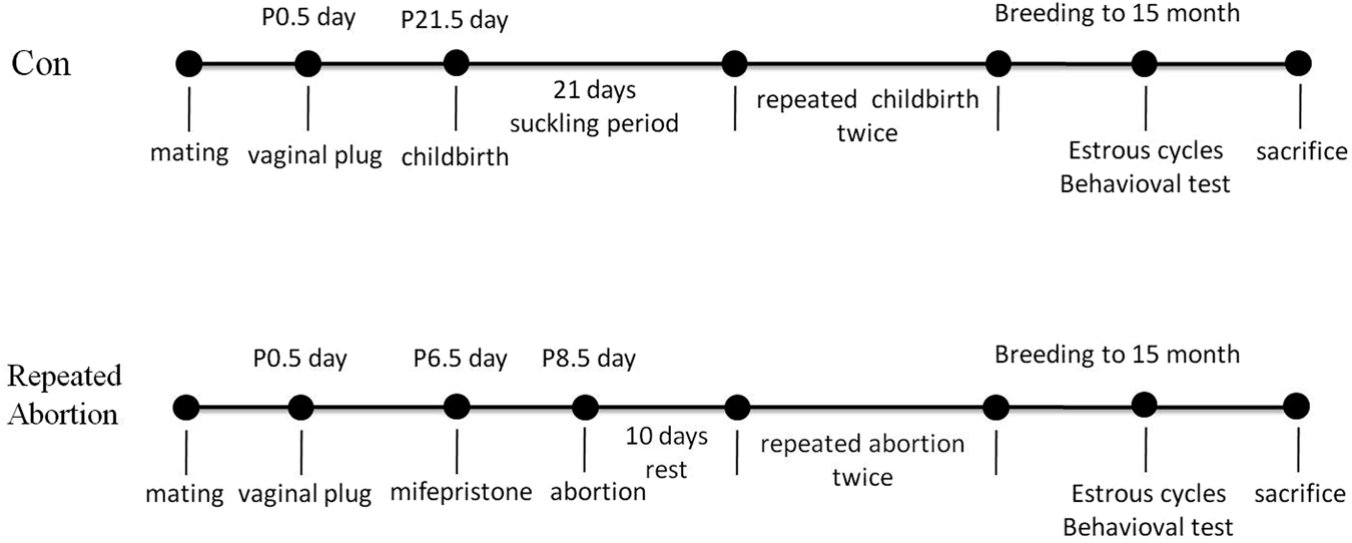
The protocol of experiment.

The pregnant mice had a normal pregnancy, a normal delivery and a normal suckling period. According to the same method, the mice were repeated pregnancy and childbirth twice after 21 days of suckling period. When breeding to 15 months, mice were proceeded behavioral test. The protocol was shown in Fig. 1.

### Repeated abortion model

The pregnant mice were administered mifepristone at the dose of 2 mg/kg on day 6.5 of pregnancy. It was determined abortion if vaginal bleeding and embryo analogue excretion within 48 hours. It would be given up follow-up tests if there were no vaginal bleeding and embryo analogue excretion within 48 hours. According to the same method, the mice were repeated abortion twice after 10 days of rest. When breeding to 15 months, mice were proceeded behavioral test. The protocol was shown in Fig. 1.

### Estrous cycle monitoring

Daily vaginal smears were collected between 10:30 to 11:30 AM from all animals when mice breeding to 15 months and continued for 14 days.

### Water maze

Mice were trained in a 1.0-m diameter open-field water maze filled with water (26 °C) and made opaque with liquid latex. Prominent extra-maze visual cues around the room remained in fixed positions throughout the experiment. During behavioral testing, mice were required to locate a hidden submerged platform (10 cm in diameter and 1.5 cm below the surface), which remained in the same position across trials for individual mice but was counterbalanced across animals. Four equally spaced points (north, south, east, and west) around the edge of the pool were used as starting positions. The mice were given four trials per day for 4 days. Trials began as the mouse was placed in the pool facing the side wall at a start position and ended once the animal found the platform. If the mouse did not find the platform within 90 s, it was guided there by hand. After 30 s on the platform, the mouse was immediately replaced in the pool at a different start position for the next trial. A video camera mounted to the ceiling directly above the center of the maze was used in conjunction with the EthoVision animal tracking system.

### Step Down Assay

Step down behavior was employed to examine memory loss by the procedure with slight modifications. The apparatus consisted of a plexiglass box with a grid floor, having a shock free zone (SFZ) (central platform) on the center of the grid floor. Electric shock (20 V AC) was delivered to the grid floor. One day prior to conducting the test, each mouse was trained to stay on the SFZ for at least 90 s; for this the animal was applied shock for 15 s every time when the mouse stepped down placing all paws on the grid floor. The process was repeated until the animal learned to stay on the SFZ for at least 90 s. Retention of memory was tested on the consecutive days by administrating treatment 30 min before the test. After the drug administration, each mouse was again placed on the SFZ, and then step down latency (SDL) and number of mistakes were observed for 5 min. A significantly increased SDL and decreased the number of mistakes compared to the vehicle control are the index of protective effect on the retrieval of memory.

### Immunohistochemistry

The immunohistochemical appearance of the ERα and ERβ was successfully optimized on paraffin section. Briefly, paraffin sections were deparaffinised and hydrated through xylene and graded alcohol series, respectively. After rinsing with water, sections were boiled in 0.1 M citric acid (pH 6.1) for 10 minutes and allowed to cool down to room temperature. Sections were washed with PBS and placed in 0.3% H_2_O_2_ to quench endogenous peroxidase activity, and washed again. Sections were incubated with normal blocking serum for 1 hour and then with anti- ERα (1:100) antibody overnight respectively. After washing, sections were incubated for 1 hour with biotinylated secondary antibody followed by incubation with a preformed complex of avidin and biotinylated peroxidase. Sections were incubated in peroxidase substrate solution (Diaminobenzidine Tetrahydrochloride, DAB) until desired stain intensity developed, rinsed with water, cleared and mounted.

### Measurement of TNF-α, IL-1β, and IL-6 levels

IL-1β, IL-6, and TNF-α levels were measured using commercially available ELISA kits.

### ELISA test for Aβ1-40

Concentrations of Aβ1-40 in CA and DG of hippocampus and cortex were determined using ELISA kit against rat Aβ1-40 (IBL, Japan).

### SHSY5Y cell cultures

SHSY5Y cells were dissociatedby trypsinization (0.25% [w/v] trypsin and 0.02% ethylene-diaminetetraacetic acid in Ca^2+^-and Mg^2+^-free Hank’s balanced salt solution) at 37 °C for 10 min, followed by gentle trituration in plating medium (h-DMEM (Dulbecco’s modified Eagle’s medium) supplemented with 10% fetal bovine serum.

### Analysis of cell viability

Cell viability was determined using an MTT assay. Cells were seeded in 96-well plates at 1 × 10^4^ cells per well and grown to 70% confluence in culture medium. The medium was replaced bymedium containing LPS (1 mg/L) or LPS + TSL (10,100 µM) for 24 h. A total of 5 g/L MTT was added to eachwell after 24 h, and the culture incubated for another 4 h at 37 °C. Then, the medium was aspirated, dye crystals weredissolved in dimethyl sulfoxide, and the absorbance was read on an ELISAplate reader using a 490 nm filter.

### Hoechst 33342 staining

After treatment with LPS (1 mg/L) or LPS + TSL (100 µM) for 24 h, the cells were fixed with 4% paraformaldehyde for 30 min at 25 °C, and then washed with pre-chilled phosphate-buffered saline three times and exposed to 10 mg/L Hoechst 33342 at room temperature in the dark for 10 min. Samples were observed under a fluorescence microscope (Nikon Optical TE2000-S).

### Real-time polymerase chain reaction (PCR)

Total RNA was extracted using a TRIzol kit. The reverse transcription reaction was performed using 2 µg of total RNA that was reverse transcribed into cDNA using oligo (dT) primer. The quantitative real-time PCR (qPCR) analysis was carried out using Taqman® one-step PCR Master Mix (Applied Biosystems, Foster City, CA). cDNA templates (2 µl) were added per 25-µl reaction with sequence-specific primers and Taqman® probes. Sequences for all target gene primers and probes were purchased commercially. The qPCR assays were carried out in triplicate on a StepOnePlus sequence detection system. The cycling conditions were 10-minute polymerase activation at 95 °C, followed by 40 cycles at 95 °C for 15 seconds and 60 °C for 60 seconds. The threshold was set above the non-template control background and within the linear phase of the target gene amplification to calculate the cycle number at which the transcript was detected. The primers used were in Table 1.

**Table 1:** Primers for real-time PCR.

Gene	Primer sequences
IL-1β	F: 5′ ACAGATGAAGTGCTCCTTCCA 3′ R: 5′ GTCGGAGATTCGTAGCTGGA 3′
IL-6	F: 5′ CTGGATTCAATGAGGAGAC 3′ R: 5′ ATTTGTGGTTGGGTCAGG 3′
TNFα	F: 5′ TTGAGGGTTTGCTACAACATGGG 3′ R: 5′ GCTGCACTTTGGAGTGATCG 3′
GAPDH	F: 5′ AAGGCTGTGGGCAAGGTCATC 3′ R: 5′ GCGTCAAAGGTGGAGGAGTGG 3′

### Western blot analysis

The proteins were separated by SDS-PAGE and electrophoretically transferred onto polyvinylidene fluoride membranes. Membranes were blocked with 5% skimmed milk for 1 h and incubated overnight at 4 °C with anti-actin and anti-HMGB1. Actin was used as a loading control. Subsequently, the membranes were incubated with the corresponding secondary antibodies, and the reaction was visualized with chemiluminescence reagents provided with an ECL kit (Bioworld) and exposed to X-ray film. The intensity of the blots was quantified with densitometry.

### Statistical analyses

The data was performed with repeated measures and two-way ANOVA followed by a Bonferroni multiple group comparison. For statistical analysis, a standard software package (SAS 10.0) was used. All data are presented as means ± SEM. Statistical significance was set at P < 0.05.

## Results

### Repeated abortion impaired working memory in old age mice

**Figure 2:**
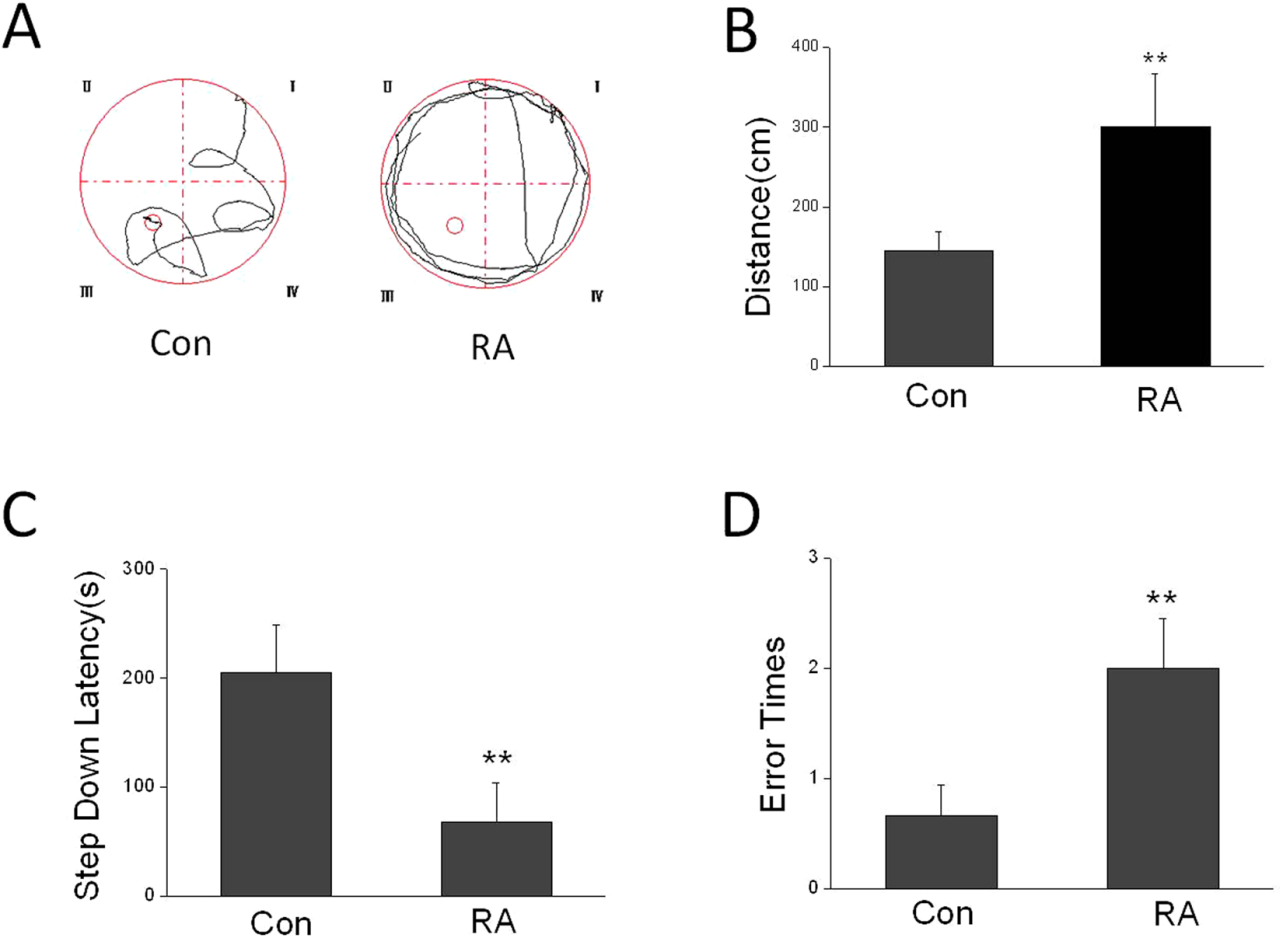
Effects of repeated abortion on learning and memory in aging. (**A**) Motion trail of the morris water maze in control mice and repeated abortion mice in aging. (**B**) Distance to a submerged platform in morris water maze. (**C**) The step down latency in step down assay. (**D**) The error times in step down assay. x ± s. ^*^P < 0.05, ^**^P < 0.01 vs. Con. n = 10.

To evaluate the potential alterations in cognitive performance due to repeated abortion during adulthood, we tested the mice at old age in relevant learning and memory tasks. We observed clear differences in 12 months old. In the Morris water maze, mice of the repeated abortion were able to significantly increase the distance to reach the platform compared to controls (Fig. 2A,B). In the jump platform experiment, mice of the repeated abortion decreased the step down latency and increased the error times (Fig. 2C,D).

### Repeated abortion increased Aβ1-40 level in CA region of hippocampus

**Figure 3:**
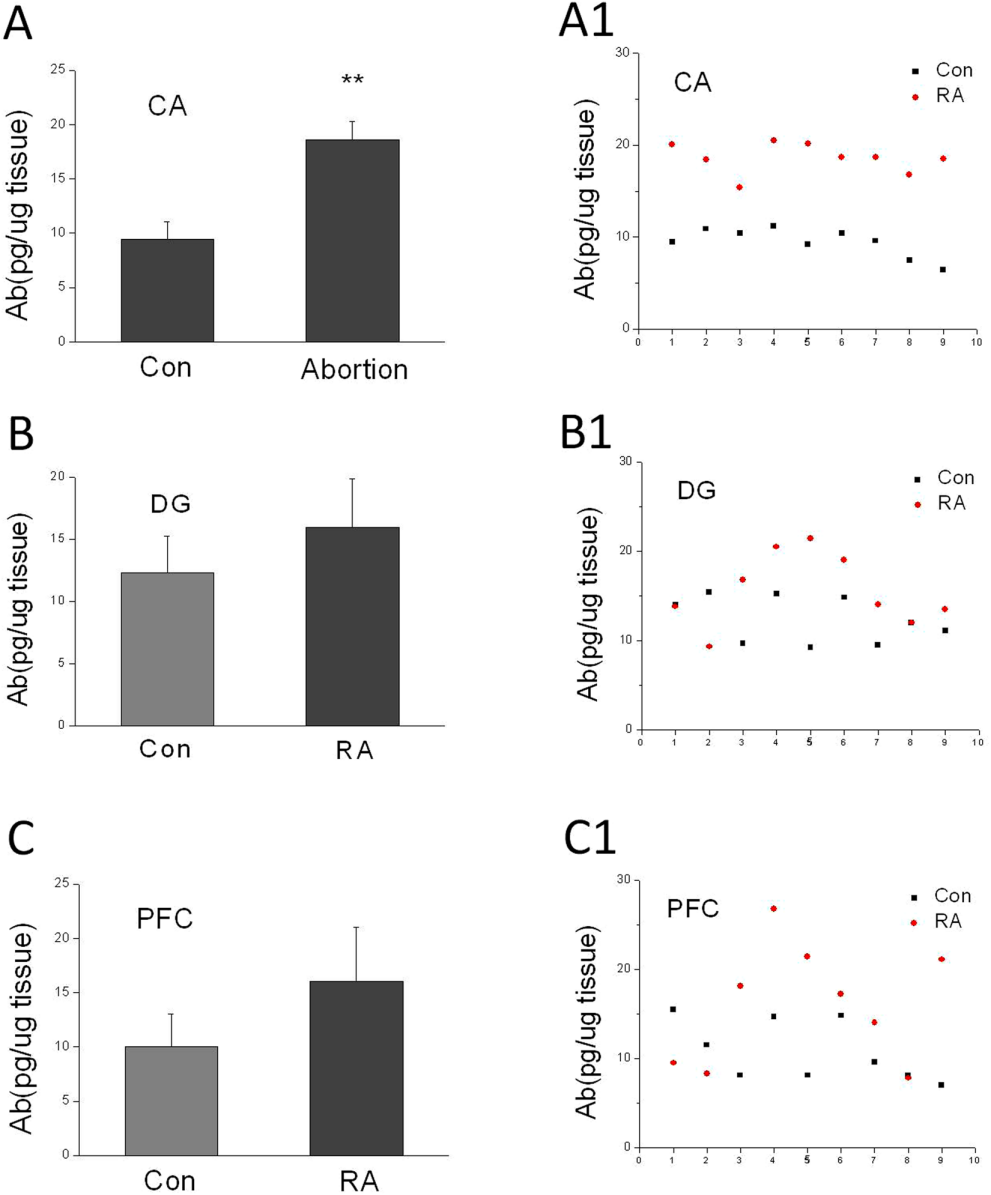
(**A**) β1-40 levels in CA (**A**,**A1**), DG (**B**,**B1**) and PFC (**C**,**C1**) subregions of hippocampus in aging mice. Repeated abortion remarkably increased the Aβ1-40 levels of in CA subregion. (**A**–**C**) Bar graph, (**A1**–**C1**) scattered plot. x ± s. ^*^P < 0.05, ^**^P < 0.01 vs. Con. n = 9.

Repeated abortion increased Aβ1-40 level in CA subregion compared with that of in control (Fig. 3A,A1, Con: 9.46 ± 1.59 vs RA: 18.59 ± 1.66; p < 0.01). There were no significant difference in DG subregion between control mice and repeated abortion mice (Fig. 3B,B1, Con: 12.32 ± 2.57 vs RA: 15.59 ± 4.06; p > 0.05). The Aβ1-40 level in PFC subregion in repeated abortion had a rising trend, but there is no difference compared with that of in control (Fig. 3C,C1, Con: 10.82 ± 3.38 vs RA: 16.02 ± 6.61; p = 0.052).

### Repeated abortion mice have uniform estrous cycles and decreased Erα expression in hypothalamus

**Figure 4:**
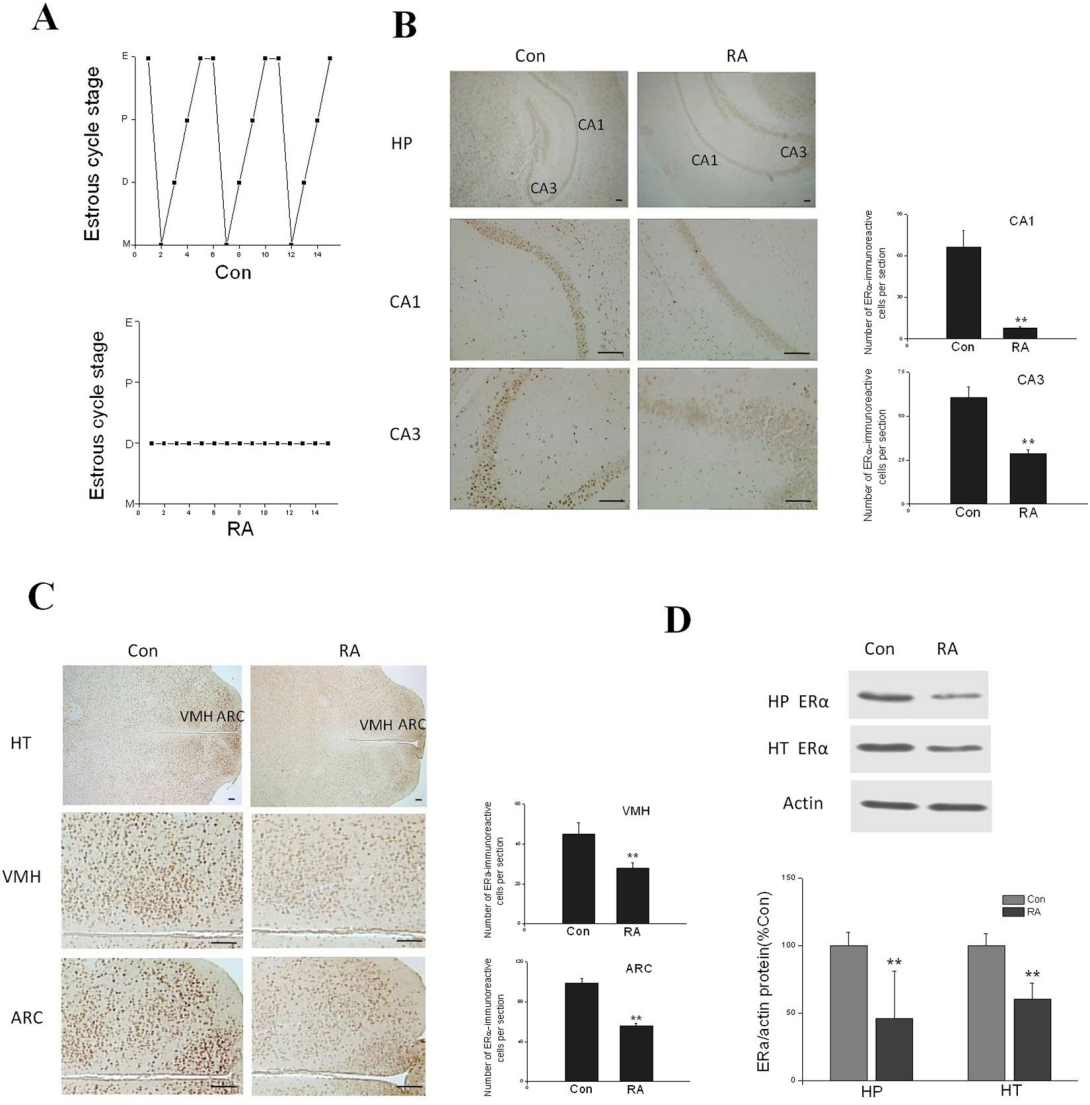
(**A**) Estrous cycles of control and repeated abortion mice in aging. D diestrus, E: estrus, M: metestrus, P: proestrus. Control mice in aging exhibited normal 4–6 day estrous cycles and repeated abortion mice became acyclic. n = 10. (**B**) Repeated abortion induced ablation of ERα expression in CA1 and CA3 subregions of hippocampus (HP). Scale bars: 100 µm. Bar chart illustrates quantification of ERα positive cells in the CA1 and CA3 subregion. (**C**) Repeated abortion induced ablation of ERα expression in VMH and ARC subregions of hypothalamus (HT). Scale bars: 100 µm. Bar chart illustrates quantification of ERα positive cells in VMH and ARC subregions. (**D**) Repeated abortion induced ERα protein expression downregulation in HP and HT in aging. x ± s. ^**^P < 0.01, ^*^P < 0.05 vs. Con. n = 6.

The estrous cycles of control and repeated abortion mice in aging were assessed by daily vaginal smear for two weeks. Control mice in aging exhibited normal 4 day estrous cycles throughout this period. In contrast, repeated abortion mice became acyclic in aging (Fig. 4A).

There were significant difference between control mice and repeated abortion mice in the number of ERα-positive neurons located in the CA1 and CA3 subregions of hippocampus. The ERα-positive neurons were suppressed in repeated abortion mice than those of in control mice in aging (Fig. 4B). Likewise, there were significant difference between control mice and repeated abortion mice in the number of ERα-positive neurons located in the VMH and ARC subregions of hypothalamus. The ERα-positive neurons were suppressed in repeated abortion mice than those of in control mice in aging (Fig. 4C). Furthermore, Repeated abortion induced ERα protein expression downregulation in HP and HT in aging (Fig. 4D).

### Repeated abortion increased mRNA and protein expression of HMGB1 in brain

**Figure 5:**
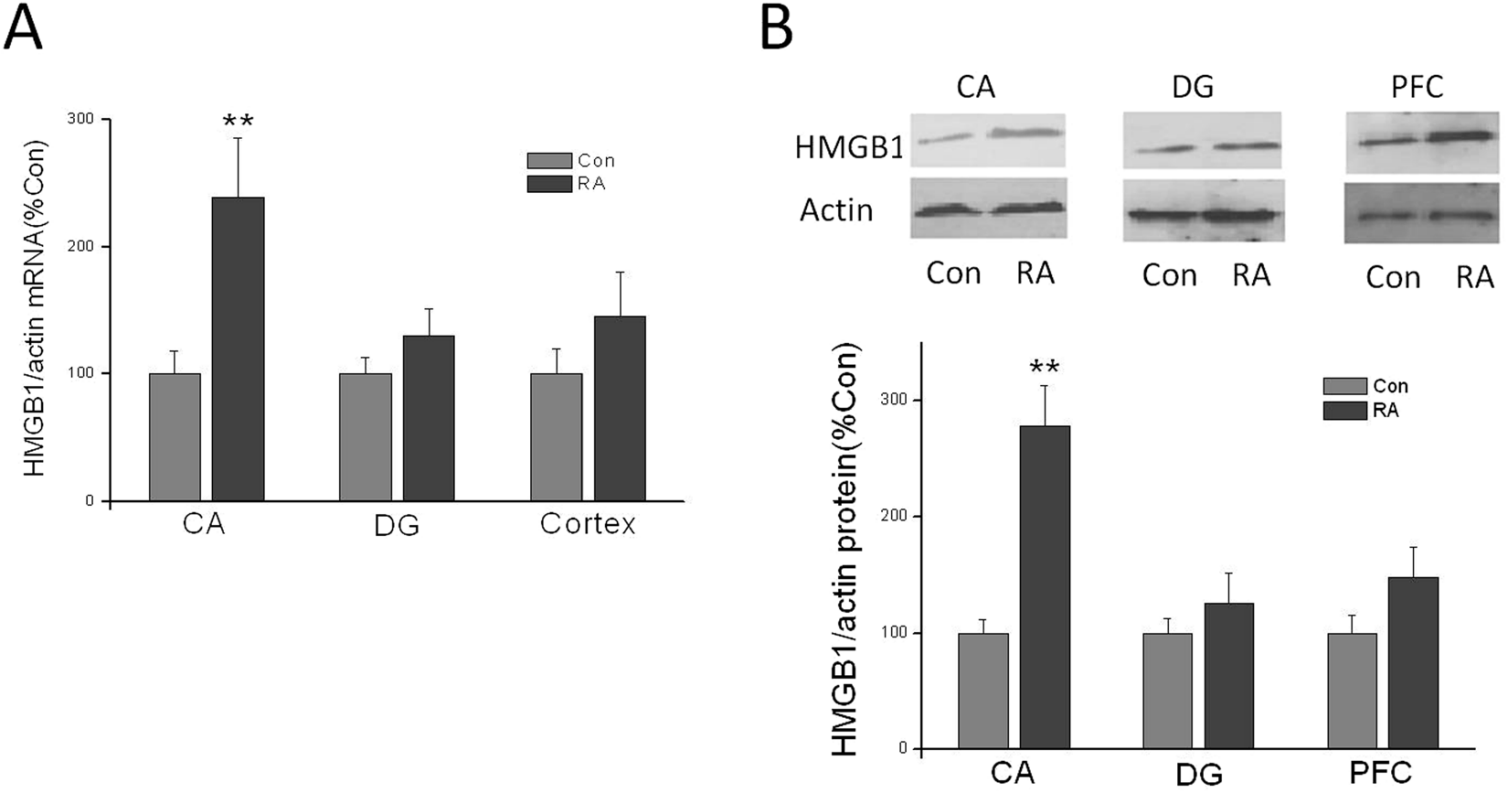
Repeated abortion induced HMGB1 mRNA and protein expression upregulation in CA, DG and PFC in aging mice. (**A**) mRNA expression of HMGB1 in CA, DG and PFC. (**B**) Western blotting estimates HMGB1 in CA, DG and PFC. x ± s. ^*^P < 0.05, ^**^P < 0.01 vs. Con. n = 4.

To examine the effects of repeated abortion-induced HMGB1 expression *in vivo*, the mRNA and protein levels of HMGB1 were determined in CA, DG and PFC. The mRNA and protein of HMGB1 in CA subregion in repeated abortion were markedly higher than those in control group. In DG and PFC region, there were no significant changes in repeated abortion mice and control mice (Fig. 5).

### Repeated abortion increased plasma levels and mRNA expression of interleukin (IL)-1β, IL-6, and tumor necrosis factor (TNF)-α in hippocampus

**Figure 6:**
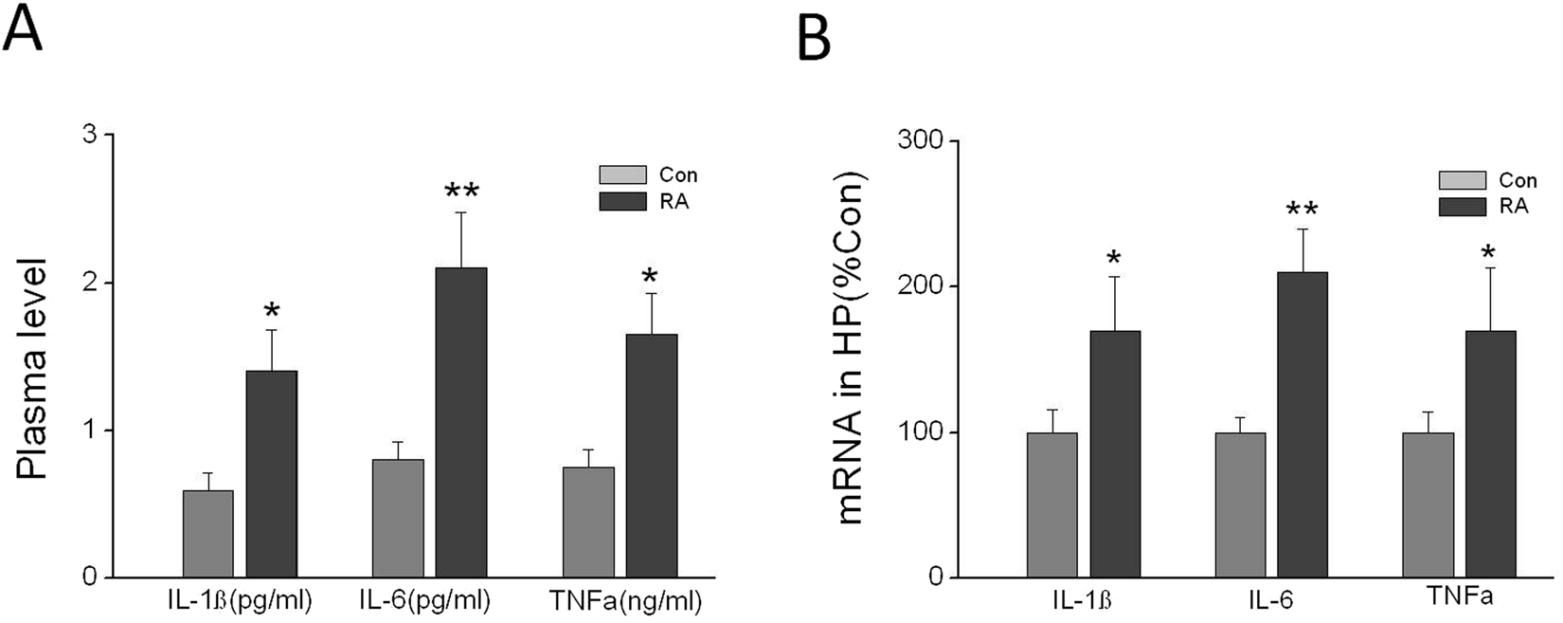
(**A**) Repeated abortion increased the plasma levels of IL-1β, IL-6 and TNFα in aging mice. n = 10. (**B**) Repeated abortion increased the mRNA expression of IL-1β, IL-6 and TNFα in hippocampus in aging mice. x ± s. ^*^P < 0.05, ^**^P < 0.01 vs. Con. n = 6.

To examine the effects of repeated abortion on inflammation *in vivo*, the plasma levels of IL-1β, IL-6, and TNF-α were determined. The results showed that the mean plasma level of IL-1β, IL-6 and TNF-α in repeated abortion were markedly higher than those in control group (Fig. 6A). Furthermore, we also measured the mRNA expression of IL-1β, IL-6, and TNF-α in hippocampus. The results showed that the relative mRNA levels of IL-1β, IL-6 and TNF-α in the repeated abortion group were much higher than those in the control group (Fig. 6B).

### MPP induced cytotoxicity in SHSY5Y cells

**Figure 7:**
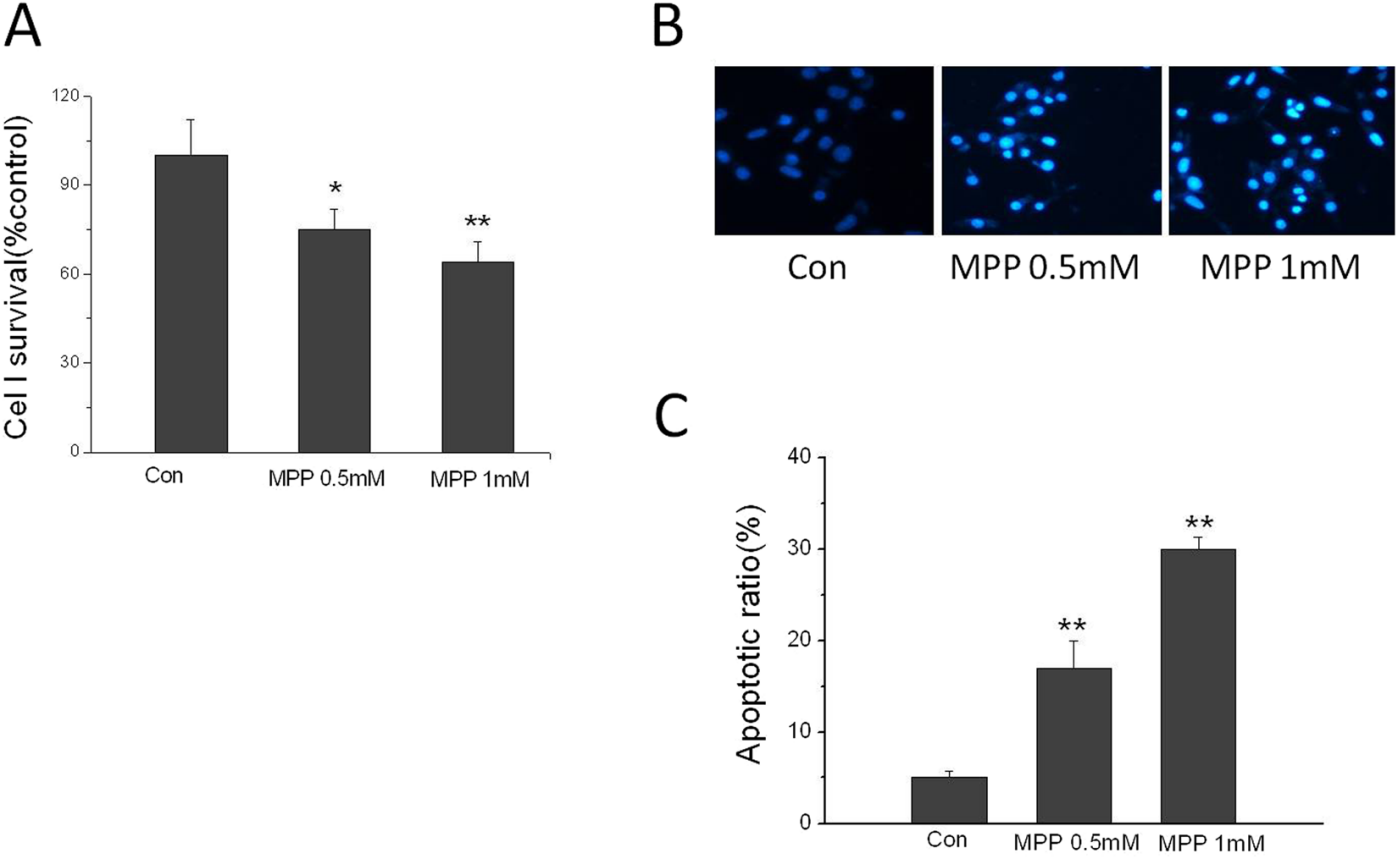
MPP-induced cytotoxicity in SH-SY5Y cells. (**A**) MPP stimulation decreased cell viability. Cell viability was assessed by MTT. (**B**) Fluorescence photomicrographs of neurons with Hoechst 33242 staining. (**C**) MPP stimulation increased cell apoptosis. x ± s. ^*^P < 0.05, ^**^P < 0.01 vs. Con. n = 4.

After incubation with MPP at the concentration of 0.5 and 1 mM, cell viability was decreased to 75% and 64%, respectively (Fig. 7A).

Hoechst 33342, which stains DNA, was used to assess changes in nuclear morphology. The nuclei in control cells appeared normal and exhibited diffuse staining of the chromatin. After exposure to 0.5 and 1 mM MPP for 24 h, neurons underwent morphologic changes typical of apoptosis, including condensed chromatin and shrunken nuclei (Fig. 7B,C).

### MPP increased the expression of HMGB1, inflammatory factor and Aβ1-40 level

**Figure 8:**
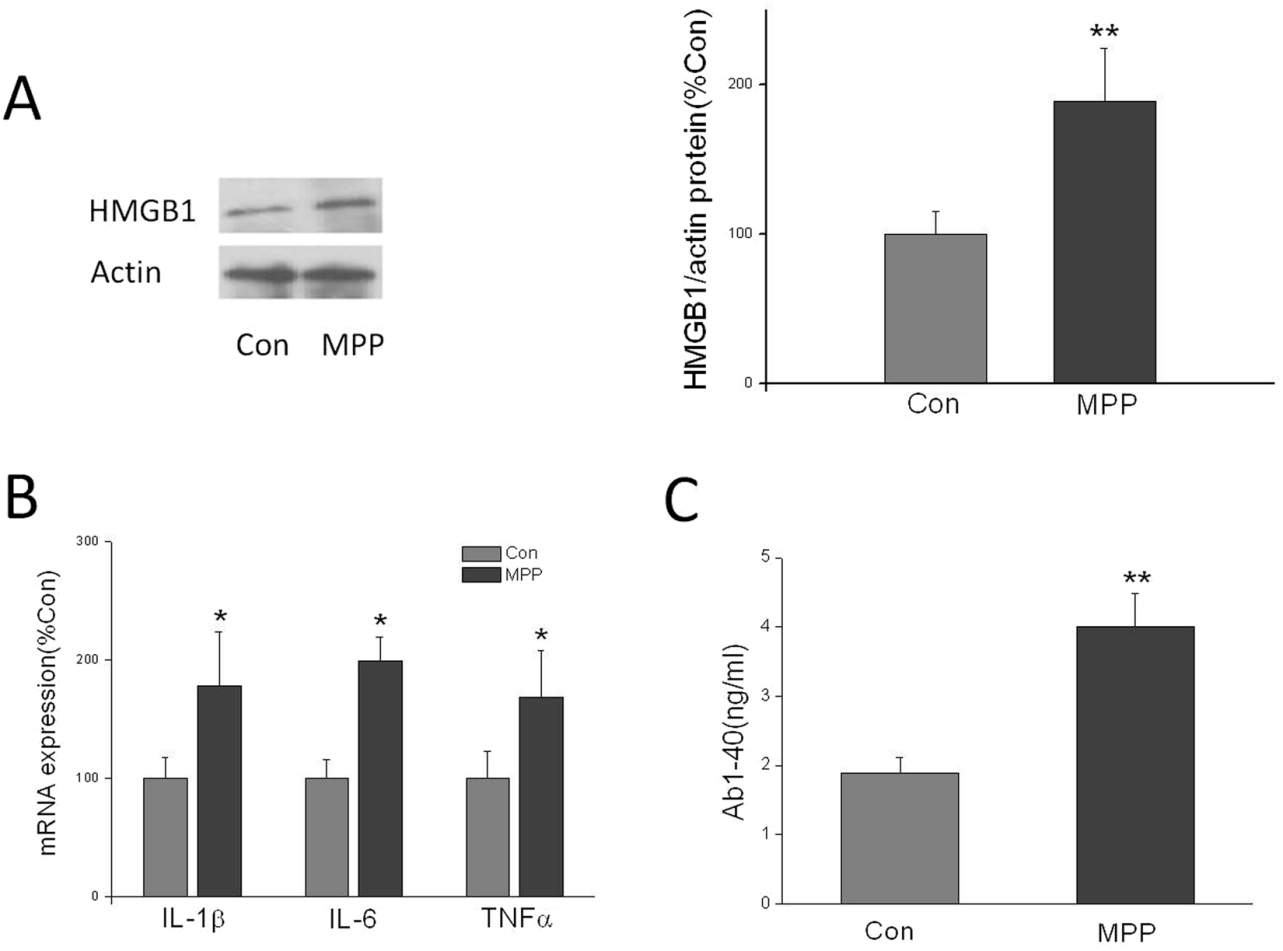
(**A**) MPP stimulated HMGB1 protein expression in SH-SY5Y cells. (**B**) MPP increased the mRNA expression of IL-1β, IL-6 and TNFα in SH-SY5Y cells. (**C**) MPP increased the Aβ1-40 level in SH-SY5Y cells. x ± s. ^*^P < 0.05, ^**^P < 0.01 vs. Con. n = 4.

We next investigated whether MPP affected the protein expression of HMGB1 (Fig. 8A). We found that the relative protein levels of HMGB1 in the MPP group were markedly higher than those in the control group. These results suggested that HMGB1 involved in the MPP-induced cytotoxicity. Likewise, the relative mRNA levels of IL-1β, IL-6 and TNF-α in the MPP group were markedly higher than those in the control group (Fig. 8B). In addition, the Aβ1-40 level in the MPP group were also markedly higher than those in the control group (Fig. 8C).

## Discussion

There is good evidence to suggest stress as a general risk factor for the development of cognitive impairment. The impact of repeated abortion during adulthood on cognitive function in the elderly has not yet been studied. In this study, we report that the effects of repeated abortion during adulthood on memory performance during aging. When the mice were 15 month old, the cognition impairment could be detected in the repeated abortion mice compared with the control mice.

In our study, we found that repeated abortion not only increased the distance in water maze, but also decreased the step down latency and increased the error times in step down assay in aging mice. Aβ is associated with learning and memory deficits. We found that there is a significant change in concentration of Aβ in different brain areas in repeated abortion mice compared with that in control mice. Our result showed that the Aβ level in CA in repeated abortion mice was much higher than that in control mice. The Aβ level in DG and cortex in repeated abortion mice had no significantly changes compared with those in control mice. Hwang found that the worsened memory impairment in the ERα knockout mice was associated with elevated Aβ level, astrogliosis and neuroinflammation, and apoptotic neuronal cell death in the brain. Furthermore, the memory impairment in the ERα knockout mice through accumulation of Aβ via reduced degradation and scavenging of Aβ by the reduced expression and activity of neprilysin^15^. In our research work, higher accumulation of Aβ in the brains was detected in repeated abortion mice.

The estrous cycle can affect cognition^16,17^. We found that the duration of estrous cycle in the control mice were regular, where as that in repeated abortion mice were disturbed. The estrous cycle is associated with approximately 2–3% changes in hippocampal volume as seen by high-resolution *ex-vivo* MRI^18,19^. Changes in hippocampal volume are, moreover, associated with a switch between hippocampal and striatal based navigation strategies. Estrous cycle regulates activation of hippocampal Akt, LIMK, and neurotrophin receptors^20^. In our work, the period of the loss of estrous cycles in aging repeated abortion group was significantly earlier than that in aging control group. The number of ERα-immunoreactive neurons and staining intensity were reduced in the hippocampus and hypothalamus in repeated abortion group. This provides direct evidence that ERα expressing cells are involved in cognition in aging mice caused by repeated abortion in adulthood. Interestingly, the impairment of learning and memory due to adulthood repeated abortion seems to be a developmental process interacting with aging, as it was not evident in young adult mice. However, this result should be interpreted with caution in future.

HMGB1 mediates this neuroinflammatory “priming” in aged animals. HMGB1 gene and HMGB1 protein expression were elevated under basal conditions in the hippocampus of aged rats^21^. Moreover, aged animals had increased HMGB1 in the CSF. HMGB1 appears to be multifunctional -a protein “for all seasons”. It is involved in replication, DNA repair, transcription and recombination within the nucleus, while acting outside the cell as a cytokine and proinflammatory mediator^22^. In our study, we found that the repeated abortion mice were accompanied by elevated mRNA and protein expression of HMGB1 and high level of IL-1β, IL-6, and TNF-α. This might be indicated the inflammation involved in the process of cognitive decline in repeated abortion mice in age^23^.

Methyl-piperidino-pyrazole (MPP) is ERα-selective antagonist. We characterized the viability and apoptosis of MPP -induced SH-SY5Y cells. Our study revealed that MPP-induced cytotoxicity in neurons was accompanied with the upregulation of HMGB1. HMGB1 could interact with steroid hormone receptors, such as ER, prior to their binding to DNA. If a subpopulation of ER is ‘chaperoned’ to its ERE/DNA by HMGB1, this ER/HMGB1 association may provide a chaperone-assisted mechanism that would increase the local concentration of HMGB1^24^. In our study, we found that MPP were accompanied by elevated expression of HMGB1, high level of IL-1β, IL-6, and TNF-α and elevated Aβ level. This might be clarified the mechanism of ERα affect cognition in repeated abortion mice.

In conclusion, our research demonstrated that the motivation and cognition injury in elderly mice caused by repeated abortion require ERα expression down-regulation in hippocampus and hypothalamus. MPP-induced cell apoptosis and increased IL-1β, IL-6, and TNF-α expression HMGB1 expression as well as Aβ level. Taken together, our results identify possible molecular mechanisms underlying cognitive impairment during aging, demonstrating the repeated abortion in adulthood on cognitive function in aged mice.
